# Distinct retrosplenial cortex cell populations and their spike dynamics during ketamine-induced unconscious state

**DOI:** 10.1371/journal.pone.0187198

**Published:** 2017-10-26

**Authors:** Grace E. Fox, Meng Li, Fang Zhao, Joe Z. Tsien

**Affiliations:** 1 Brain and Behavior Discovery Institute and Department of Neurology, Medical College of Georgia, Augusta University, Augusta, GA, United States of America; 2 The Brain Decoding Center, Banna Biomedical Research Institute, Xi-Shuang-Ban-Na Prefecture, Yunnan Province, China; University of Wisconsin-Milwaukee, UNITED STATES

## Abstract

Ketamine is known to induce psychotic-like symptoms, including delirium and visual hallucinations. It also causes neuronal damage and cell death in the retrosplenial cortex (RSC), an area that is thought to be a part of high visual cortical pathways and at least partially responsible for ketamine’s psychotomimetic activities. However, the basic physiological properties of RSC cells as well as their response to ketamine *in vivo* remained largely unexplored. Here, we combine a computational method, the Inter-Spike Interval Classification Analysis (ISICA), and *in vivo* recordings to uncover and profile excitatory cell subtypes within layers 2&3 and 5&6 of the RSC in mice within both conscious, sleep, and ketamine-induced unconscious states. We demonstrate two distinct excitatory principal cell sub-populations, namely, high-bursting excitatory principal cells and low-bursting excitatory principal cells, within layers 2&3, and show that this classification is robust over the conscious states, namely quiet awake, and natural unconscious sleep periods. Similarly, we provide evidence of high-bursting and low-bursting excitatory principal cell sub-populations within layers 5&6 that remained distinct during quiet awake and sleep states. We further examined how these subtypes are dynamically altered by ketamine. During ketamine-induced unconscious state, these distinct excitatory principal cell subtypes in both layer 2&3 and layer 5&6 exhibited distinct dynamics. We also uncovered different dynamics of local field potential under various brain states in layer 2&3 and layer 5&6. Interestingly, ketamine administration induced high gamma oscillations in layer 2&3 of the RSC, but not layer 5&6. Our results show that excitatory principal cells within RSC layers 2&3 and 5&6 contain multiple physiologically distinct sub-populations, and they are differentially affected by ketamine.

## Introduction

Ketamine, a phencyclidine derivative and non-competitive N-methyl-D-aspartate receptor (NMDAR) antagonist, was first used in clinical settings because of its ability to produce potent anesthesia and analgesia, and more recently, it has been used to treat chronic pain and depression [1–5]. Dissociative anesthesia produced as a result of ketamine treatment is thought to be a result of reduced activation in thalamocortical structures and increased activity in the limbic system [2]. Ketamine use is also associated with post-operative hallucinations, vivid dreams, and delusions. Furthermore, the psychotropic effects of ketamine range from dissociation and depersonalization to psychotic experiences [6–8]. Interestingly, at sub-anesthetic doses, ketamine impairs semantic and episodic memory [8–13]. These effects are thought to be due, at least in part, to NMDAR antagonism by ketamine [13]. Despite the widespread use of ketamine in both clinical and recreational settings, characterization of the dynamic activity patterns of neurons in response to ketamine is limited.

Here, we set out to investigate the *in vivo* response patterns of neurons within the retrosplenial cortex (cortex), a region suggested to be responsible for the psychotomimetic activities of ketamine [14,15]. In humans, sub-anesthetic doses of ketamine induce in 14C-2-deoxyglucose (2-DG) uptake in the RSC and increase functional connectivity between the posterior hippocampus and the RSC [16,17]. In rodents, ketamine has been shown to cause neuronal damage [14,15]. Interestingly, sub-anesthetic ketamine doses lead to increased c-Fos expression and dopamine release in the RSC [18,19]. The RSC is a large midline structure with dense, reciprocal connections to select thalamic nuclei, prefrontal cortex, and the hippocampal formation [20–22]. Given these connections, it is not surprising that the RSC has been shown to be involved in many memory-related processes [23,24]. Indeed, the RSC has been shown to play an important role in the consolidation, storage, and retrieval of memories [24–39]. Additionally, the RSC is an important contributor to spatial cognition, which is likely related to its role in representing contexts [23,40–44]. Importantly, the neuronal populations within the RSC and their *in vivo* physiological properties, especially as they related to ketamine, remain to be investigated.

Recently, we have described a novel computational method that allows for the discovery of discrete cell sub-populations within *in vivo* neural datasets [45]. This approach, Inter-Spike-Interval Classification Analysis (ISICA), provided an invariant classification of both dopaminergic neurons from the ventral tegmental area and hippocampal CA1 excitatory principal cells [45]. Importantly, this classification remained invariant over multiple distinct brain states, including ketamine-induced anesthesia [45]. Here, using our ISICA computational classification method, we investigated neural activity datasets recorded from layers 2&3 and layers 5&6 on the RSC in freely behaving mice during quiet awake and two unconscious states, namely, sleep and ketamine-induced anesthesia.

## Results

**Fig 1:**
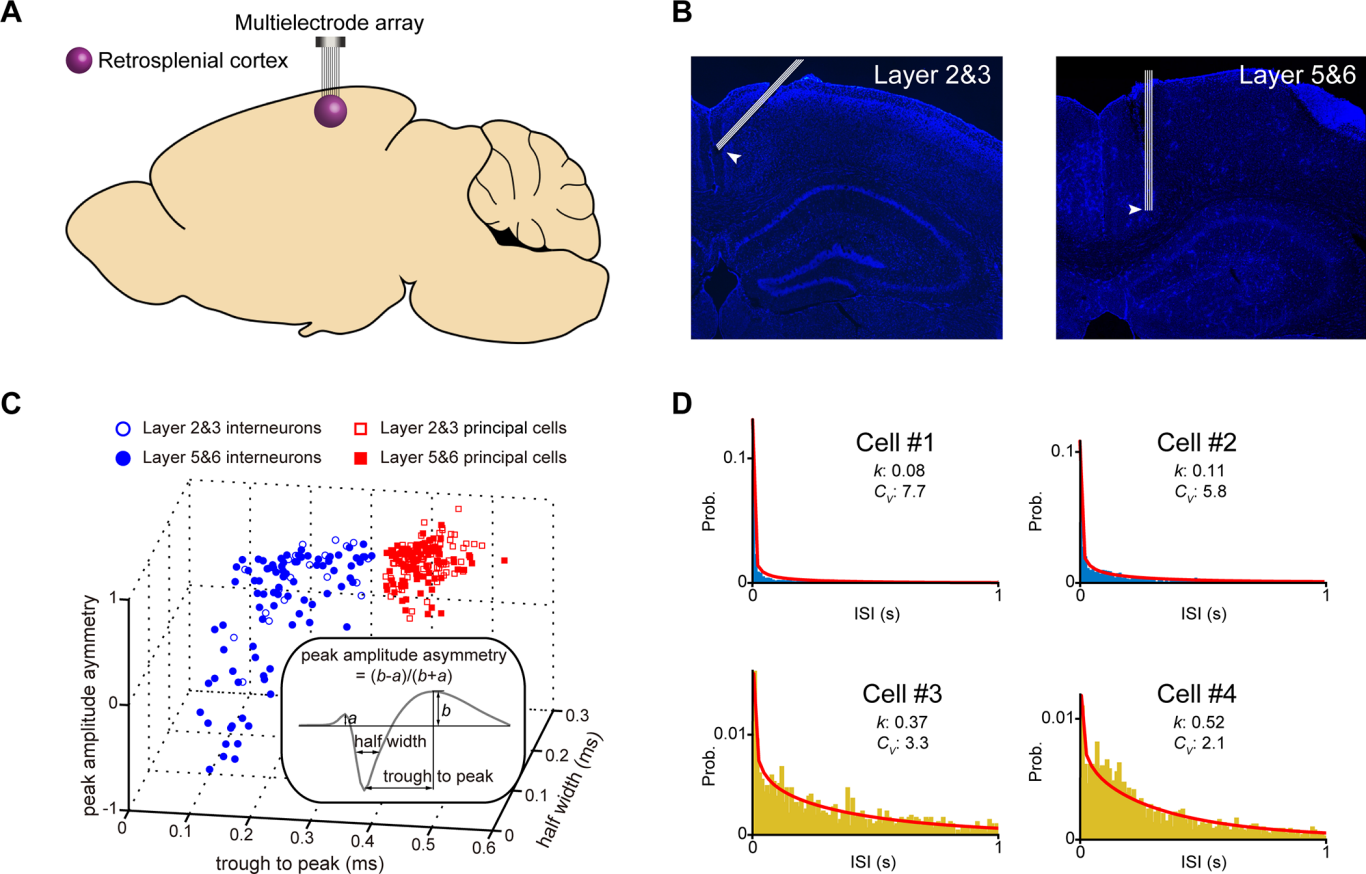
Neuronal population recording in retrosplenial cortex. **(A)** Neural activity pattern in the retrosplenial cortex was recorded using a multielectrode array. **(B)** Positions of tetrode arrays in layer 2&3 and layer 5&6 in the RSC. **(C)** Classification of putative excitatory principal vs. inhibitory RSC cells based on their spike waveform. **(D)** Neural activity patterns of four RSC principal cells showed massive variations, and these variations could be well described by *k* and *c*_*v*_. Red curves were the probability distribution function of Gamma distribution for each neuron.

We recorded neural spike activity from the layers 2&3 and layers 5&6 of the RSC in freely behaving mice during quiet awake and sleep periods, as shown in Fig 1A and 1B. The well-separated neurons were assessed by “Isolation Distance” and “*L*_*ratio*_”. In the present analysis, neurons whose “Isolation Distance” >15 and “*L*_*ratio*_” <0.7 were selected for further analysis. Isolated units (215 well-separated neurons recorded from RSC layer 2&3 of nine mice and 262 well-separated neurons recorded from RSC layer 5&6 of 13 mice) were classified as either putative excitatory principal cells or interneurons based on their spike waveforms (see Fig 1C). As shown in the subplot of Fig 1C, three waveform features were employed for the classification, that is, trough to peak, half width, and peak amplitude asymmetry. Based on these three waveform features, well-separated neurons showed clear classifications for both layers 2&3 and layers 5&6 of RSC.

As shown in Fig 1D, we employed two features, shape parameter *k* of Gamma distribution and coefficient of variation *c*_*v*_, to describe the inter-spike intervals (ISI) of RSC excitatory principal cells, the diversity of these ISIs indicated that there may be subtypes in neuron population. We then applied the Inter-Spike Interval Classification Analysis (ISICA, for short) approach to study the neuronal subtype profiles of putative excitatory principal cells in layers 2&3 and layers 5&6 of RSC.

### Profiling of RSC cortex layers 2&3 principal cell subtypes during the quiet awake and sleep periods

**Fig 2:**
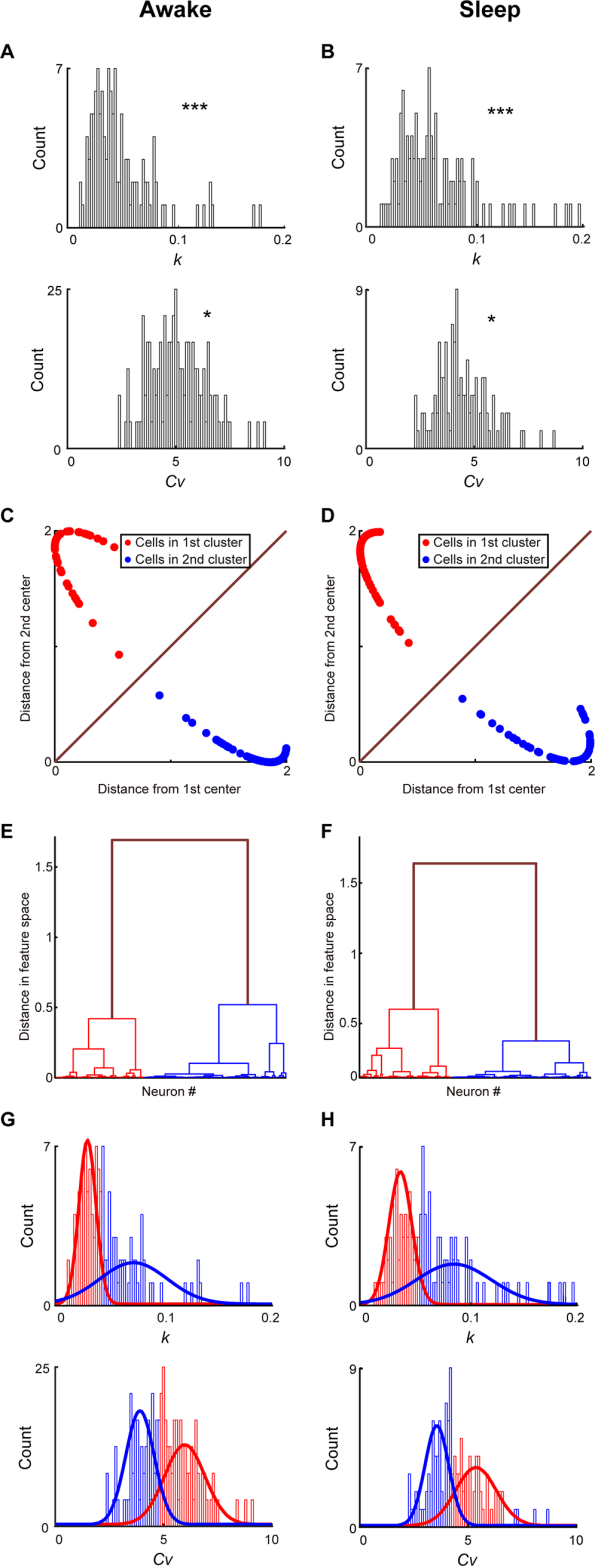
Profiling RSC layers 2&3 principal cells based on neural activity patterns during the quiet awake and sleep periods. **(A)** Distributions of *k* and *Cv* under the quiet awake state. *p* values from the D’Agostino and Pearson omnibus normality test indicated that there are discrete sub-populations within RSC layer 2&3 principal cell population. **(B)** Distributions of *k* and *Cv* during the sleep state. *p* values from the D’Agostino and Pearson omnibus normality test indicated that there are discrete sub-populations within RSC layer 2&3 principal cell population. **(C)** Distances from two cluster centers revealed a significant separation of two RSC layers 2&3 principal cell subtypes during the quiet awake period. **(D)** Distances from two cluster centers revealed a significant separation of two RSC layers 2&3 principal cell subtypes during the sleep period. **(E)** A hierarchical clustering analysis showed that the inter-cluster distance of two clusters uncovered during the quiet awake period was significantly higher than the intra-cluster distance. **(F**) A hierarchical clustering analysis showed that the inter-cluster distance of two clusters uncovered during the sleep period was significantly higher than the intra-cluster distance. In **A** and **B**, *P < 0.05, ***P < 0.001. (G) Distributions of *k* and *Cv* for two RSC layer 2&3 principal cell subtypes and their Gaussian mixture models during the quiet awake state. (H) Distributions of *k* and *Cv* for two RSC layer 2&3 principal cell subtypes and their Gaussian mixture models during the sleep state.

We first examined spike dynamics and ISI profiles of RSC principal cells in layers 2&3. The neural datasets were obtained from two behavioral states; namely, the quiet awake period and the sleep period. Among 215 well-separated neurons recorded for nine mice, a total of 138 RSC layer 2&3 putative excitatory principal cells were used for the present analysis of extracting ISI features. If these excitatory principal cells were indeed separate neuronal subtypes, we would expect to observe multimodal distributions of their properties, compared with unimodal distributions for a single neuronal class. As shown in Fig 2A and 2B, ***p*** values of the D’Agostino and Pearson omnibus normality tests showed that both *k* and *c*_*v*_ were not unimodally distributed under quiet awake (Fig 2A, *k*: ***p***<1E-14, *c*_*v*_: ***p*** = 0.043) and sleep states (Fig 2B, *k*: ***p*** = 0, *c*_*v*_: ***p*** = 0.045), suggesting that there were multiple sub-populations of RSC layer 2&3 principal cells. Our ISICA analyses suggested two well-separated, RSC principal cell sub-populations during the quiet awake (Fig 2C and 2E) and sleep (Fig 2D and 2F) periods. Fig 2C and 2D showed the distributions of two RSC principal cell subtypes in the 3D space created by *k*, *c*_*v*_, and mean firing rates. The numbers of RSC principal cells in these two distinct clusters were 57 (red dots in Fig 2E and 2F) and 81 (blue dots in Fig 2E and 2F), respectively. No significant difference was observed in the waveform of these two subtypes (***p*** = 0.567, two-sample *t*-test). Also, we used a bootstrap analysis to verify that the data were indeed best represented by two clusters. The hierarchical clustering analysis further showed that the inter-cluster distance of two clusters was significantly higher than the intra-cluster distance during the quiet awake (Fig 2G, ***p***<1E-57, two-sample *t*-test) and sleep (Fig 2H, ***p***<1E-61, two-sample *t*-test) periods. These results provided strong evidence for the existence of two distinct principal cell sub-populations in the layers 2&3 of RSC.

**Fig 3:**
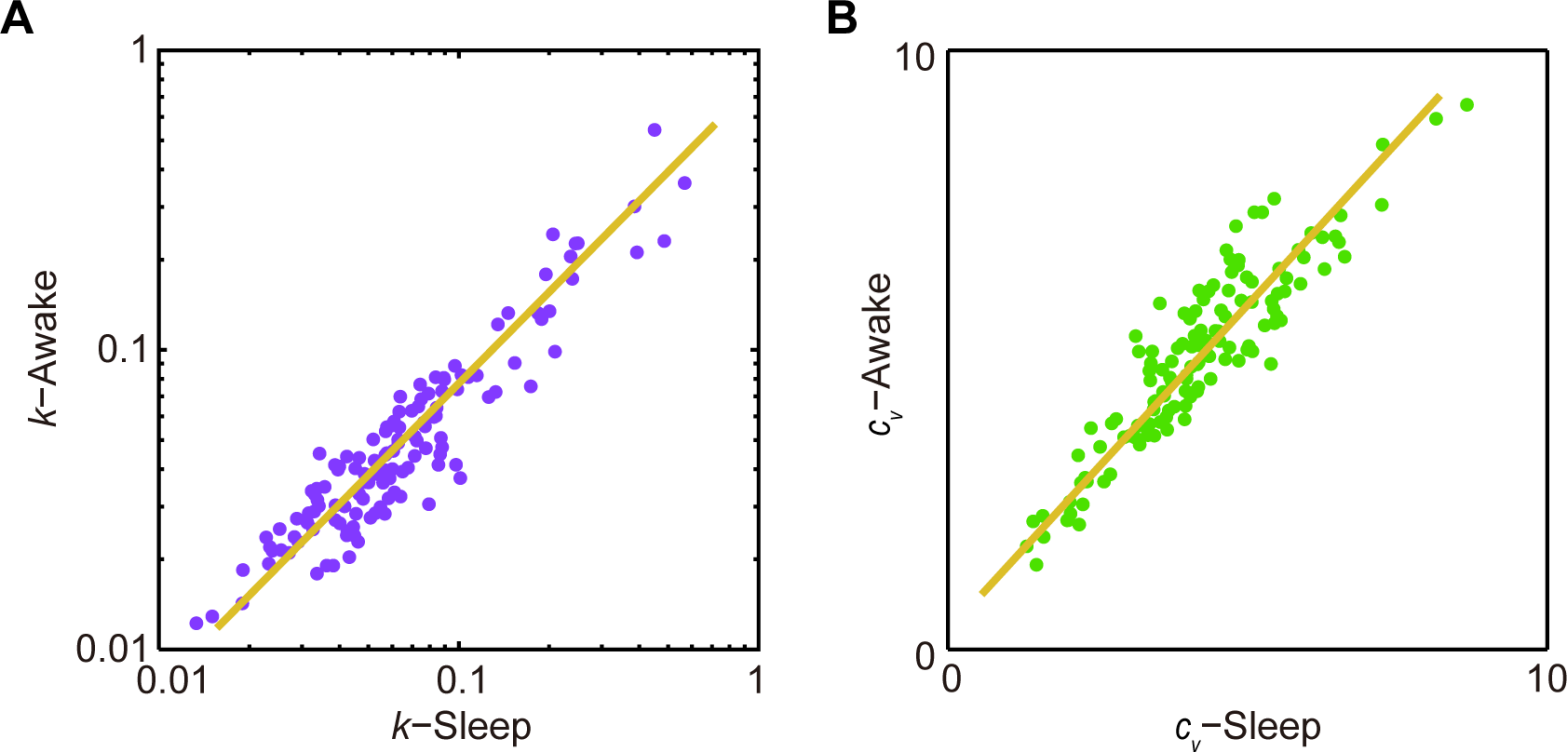
*k* and *c*_*v*_ of RSC layers 2&3 principal cells were robust across different brain states. **(A**) *k* of RSC layers 2&3 principal cells showed linear relation under the quiet awake and sleep states. **(B)** *c*_*v*_ of RSC layers 2&3 principal cells showed linear relation under the quiet awake and sleep states.

Most importantly, we observed that individual memberships of these two clusters uncovered under the sleep state were in complete agreement with those identified using the spike activity patterns recorded from the quiet awake state. In fact, *k* and *c*_*v*_ measured under these two states remained closely or proportionally matched, almost in a linear relation with linear correlation coefficients of 0.92 and 0.93 for *k* and *c*_*v*_ between the two states, respectively (Fig 3A and 3B). Taken together, these analyses suggested that ISICA-based cell classification remained robust and invariant over different brain states.

**Fig 4:**
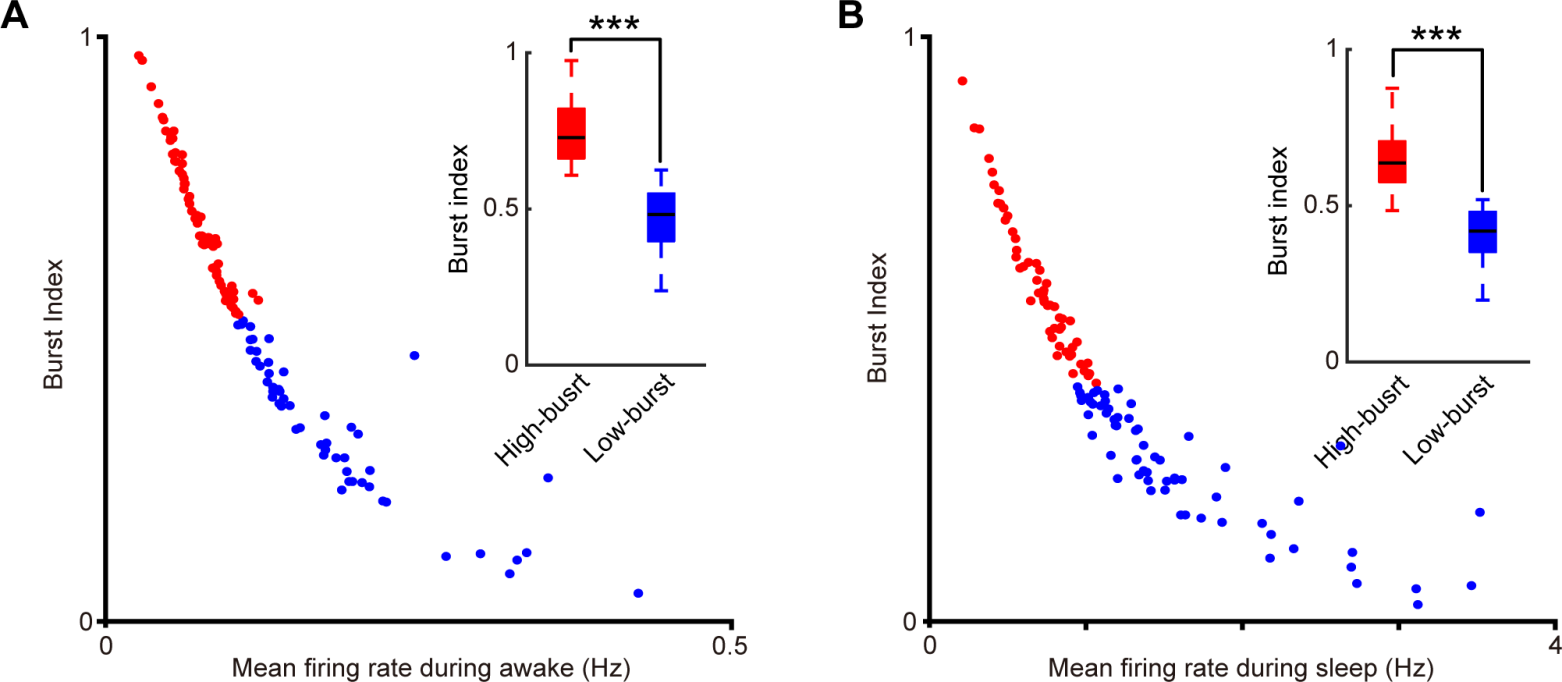
The burst index and the mean firing rate of two RSC layers 2&3 principal cell sub-populations were in negative correlation during the quiet awake and sleep periods. **(A)** The quiet awake period. **(B)** The sleep period. Subplots showed the boxplots of burst indexes of two sub-populations. Error bars indicate s.e.m.; ***P < 0.001.

We then measured the relationship between the burst index and the mean firing rate of these two-layer 2&3 principal cell sub-populations during the quiet awake and sleep states. Burst index is defined as the ratio of bursting ISIs (the ISI shorter than 10 ms) to all ISIs. We observed that the burst index and the mean firing rate of these two RSC pyramidal cell sub-populations were in negative correlation during the quiet awake (Fig 4A) and sleep (Fig 4B) states. Furthermore, we found that these two RSC principal cell subtypes indeed showed significant differences in burst indexes under the quiet awake state (0.748±0.011 vs. 0.469±0.014, ***p***<1E-30 two-sample *t*-test, the subplot in Fig 4A) and the sleep state (0.655±0.013 vs. 0.412±0.0011, ***p***<1E-27, two-sample *t*-test, the subplot in Fig 4B), suggesting that the ISICA method is highly sensitive to distinguish different bursting cells. For convenience in this study, we termed these two subgroups of RSC principal cells, identified by this ISICA method, as the high-bursting RSC principal cells and low-bursting RSC principal cells, respectively.

### Profiling of RSC layers 5&6 excitatory principal cell subtypes during the quiet awake and sleep periods

**Fig 5:**
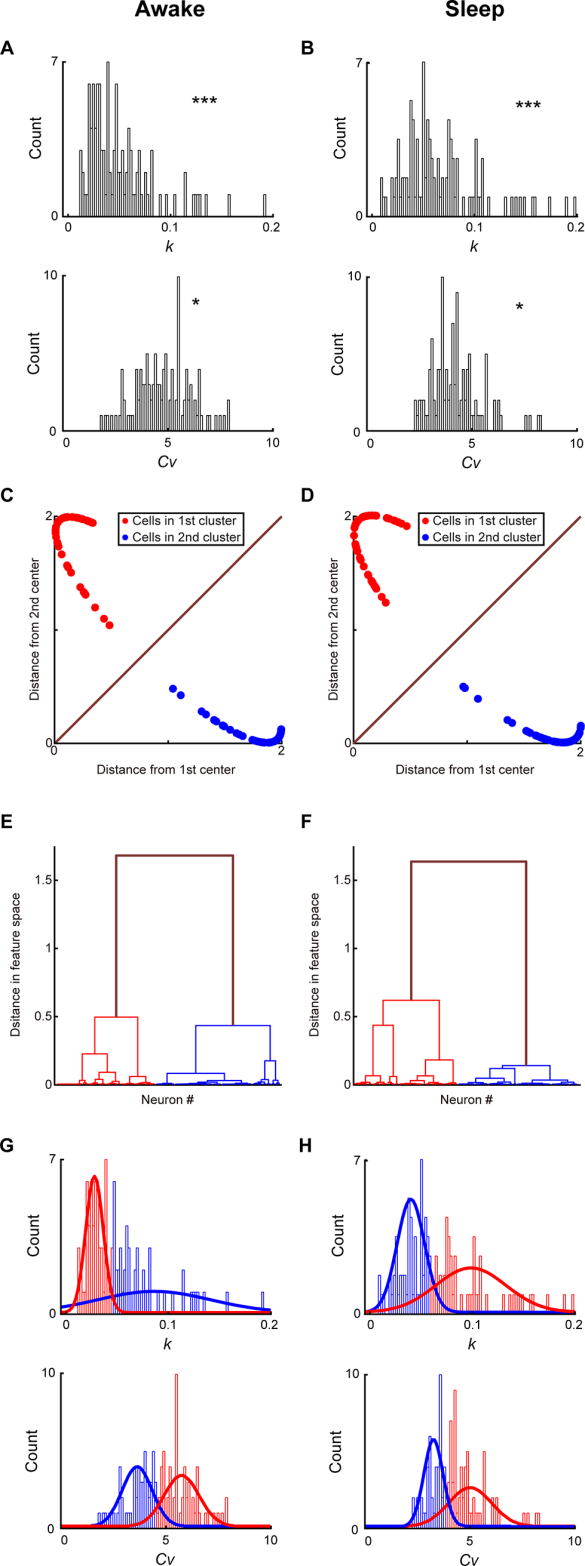
Profiling RSC layers 5&6 principal cells based on neural activity patterns during the quiet awake and sleep periods. **(A)** Distributions of *k* and *Cv* under the quiet awake state. *p* values from the D’Agostino and Pearson omnibus normality test indicated that there are discrete sub-populations within RSC layer 5&6 principal cell population. **(B)** Distributions of *k* and *Cv* during the sleep state. *p* values from the D’Agostino and Pearson omnibus normality test indicated that there are discrete sub-populations within RSC layer 5&6 principal cell population. **(C)** Distances from two cluster centers revealed a significant separation of two RSC layers 5&6 principal cell subtypes during the quiet awake period. **(D)** Distances from two cluster centers revealed a significant separation of two RSC layers 5&6 principal cell subtypes during the sleep period. **(E)** A hierarchical clustering analysis showed that the inter-cluster distance of two clusters uncovered during the quiet awake period was significantly higher than the intra-cluster distance. **(F**) A hierarchical clustering analysis showed that the inter-cluster distance of two clusters uncovered during the sleep period was significantly higher than the intra-cluster distance. In **A** and **B**, *P < 0.05, ***P < 0.001. (G) Distributions of *k* and *Cv* for two RSC layer 5&6 principal cell subtypes and their Gaussian mixture models during the quiet awake state. (H) Distributions of *k* and *Cv* for two RSC layer 5&6 principal cell subtypes and their Gaussian mixture models during the sleep state.

**Fig 6:**
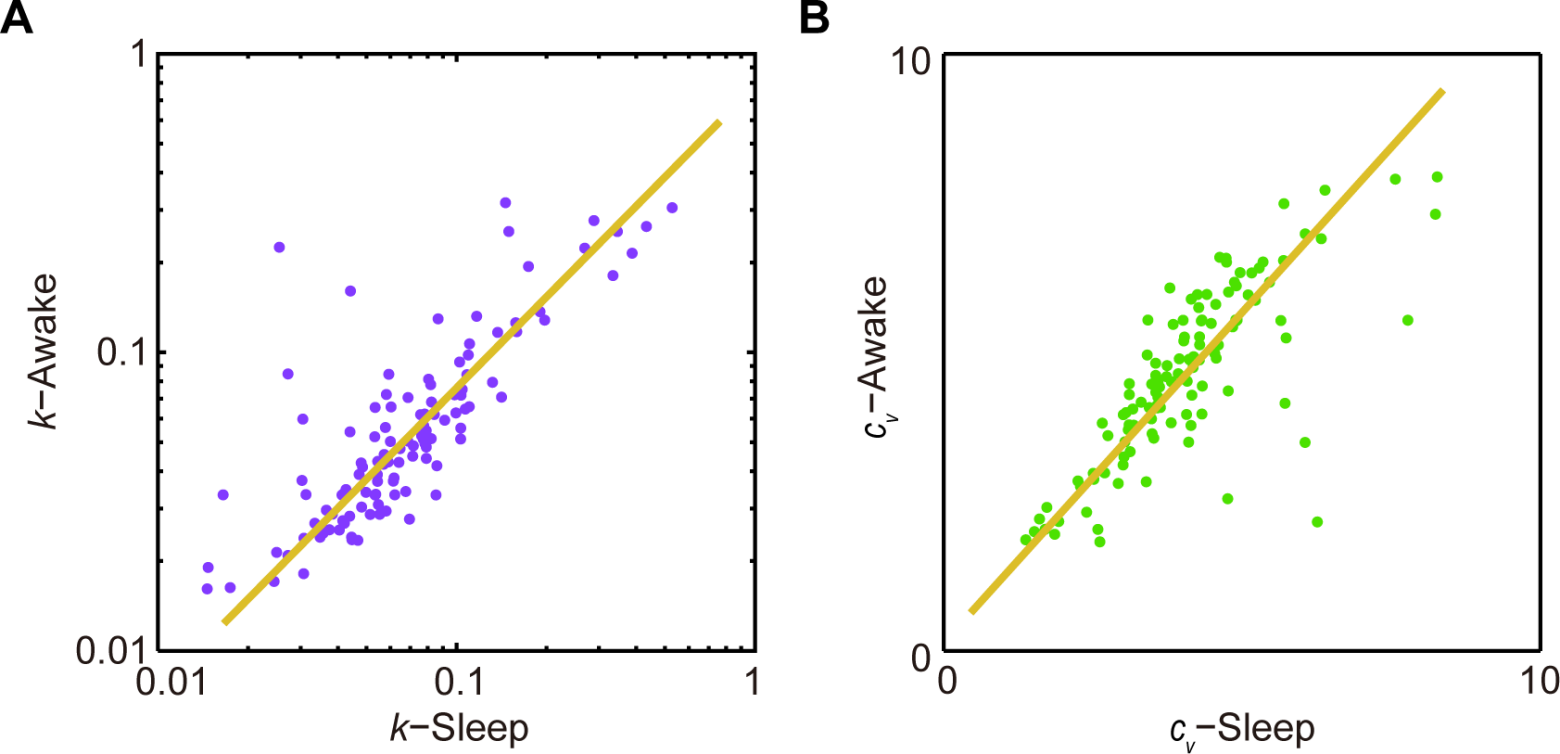
*k* and *c*_*v*_ of RSC layers 5&6 principal cells were robust across different brain states. **(A**) *k* of RSC layers 5&6 principal cells showed linear relation under the quiet awake and sleep states for. **(B)** *c*_*v*_ of RSC layers 5&6 principal cells showed linear relation under the quiet awake and sleep states.

We then analyzed the principal cells recorded from layers 5&6 of the RSC region of freely behaving mice during the quiet awake and sleep periods. Among 262 well-separated neurons recorded for 13 mice, a total of 123 RSC layers 5&6 excitatory principal cells were recorded and analyzed during the quiet awake and sleep periods. As shown in Fig 5A and 5B, ***p*** values of the D’Agostino and Pearson omnibus normality tests showed that both *k* and *c*_*v*_ were not unimodally distributed under quiet awake (Fig 5A, *k*: ***p*** = 0, *c*_*v*_: ***p*** = 0.039) and sleep state (Fig 5B, *k*: ***p*** = 0, *c*_*v*_: ***p*** = 0.012), suggesting that there were multiple sub-populations of RSC layer 5&6 principal cells. Two well-separated clusters were identified by the ISICA analysis during the quiet awake as well as sleep periods (Fig 5C and 5D), with 51 and 72 RSC principal neurons in each cluster (Fig 5E and 5F). No significant difference was observed in the waveform of these two subtypes (***p*** = 0.410, two-sample *t*-test). The hierarchical clustering analysis further proved that the inter-cluster distance of two clusters was significantly higher than the intra-cluster distance during the quiet awake (Fig 5G, ***p***<1E-57, two-sample *t*-test) and sleep (Fig 5H, ***p***<1E-59, two-sample *t*-test) periods. We also found that *k* and *c*_*v*_ measured under these two states remained highly consistent, and the linear correlation coefficients of *k* and *c*_*v*_ between the two states were 0.90 and 0.92, respectively (Fig 6A and 6B). Therefore, the ISICA method uncovered two distinct major subtypes of RSC layers 5&6 principal cells.

**Fig 7:**
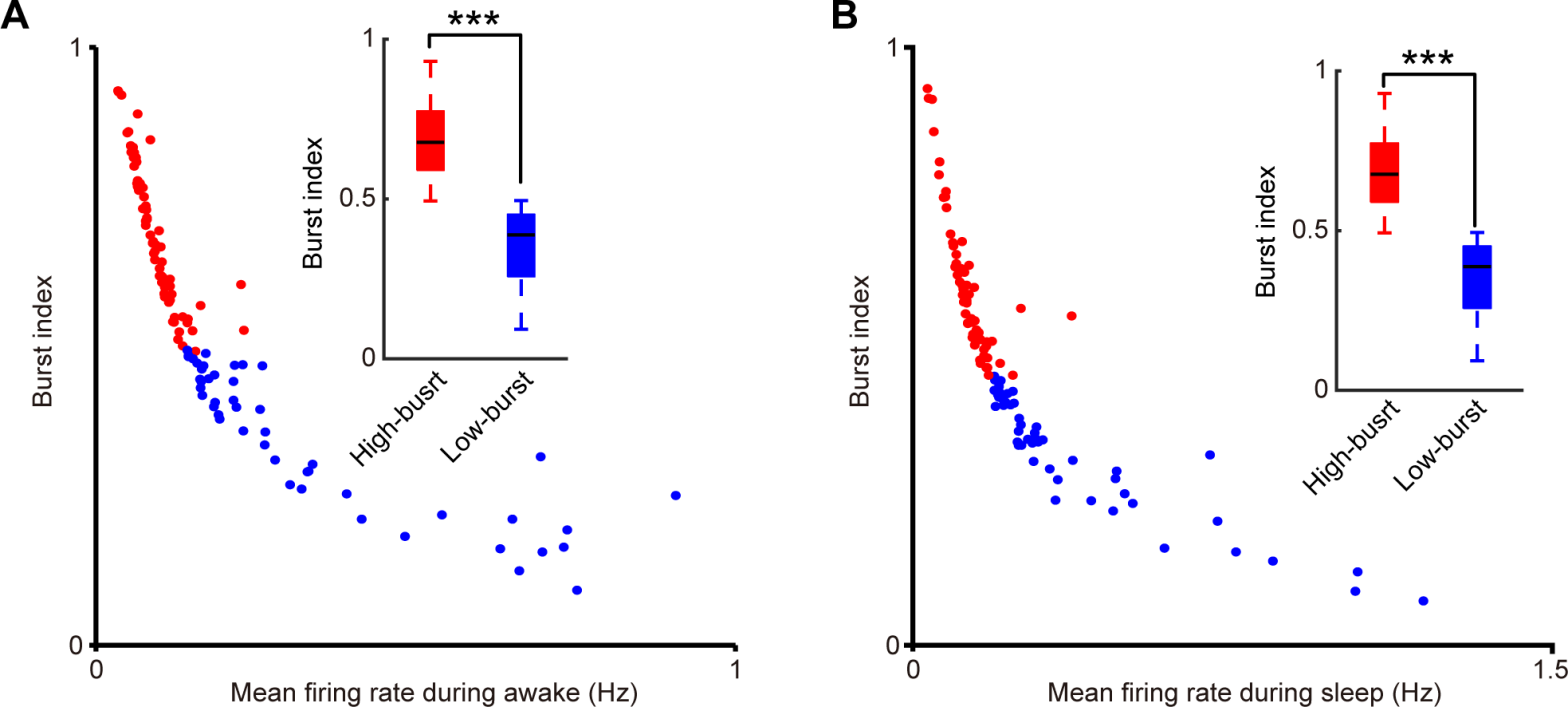
The burst index and the mean firing rate of two RSC layers 5&6 principal cell sub-populations were in negative correlation during the quiet awake and sleep periods. **(A)** The quiet awake period. **(B)** The sleep period. Subplots showed the boxplots of burst indexes of two sub-populations. Error bars indicate s.e.m.; ***P < 0.001.

Similar to the RSC layers 2&3 principal cells, the burst index and the mean firing rate of these two RSC layers 5&6 principal cell sub-populations showed negative correlation during the quiet awake (Fig 7A) and sleep (Fig 7B) periods. And these two RSC layers 5&6 principal cell sub-populations had significant differences in burst indexes under the quiet awake state (0.687±0.014 vs. 0.317±0.021, ***p***<1E-29, two-sample *t*-test, see the subplot in Fig 7A) and the SWS state (0.595±0.014 vs. 0.299±0.018, ***p***<1E-24, two-sample *t*-test, see the subplot in Fig 7B). Taken together, the ISICA analysis also uncovered the high-bursting RSC principal cells and low-bursting RSC principal cells in layers 5&6 of RSC.

### Cross-correlation analysis of layers 2&3 and 5&6 RSC excitatory principal cell subtypes

**Fig 8:**
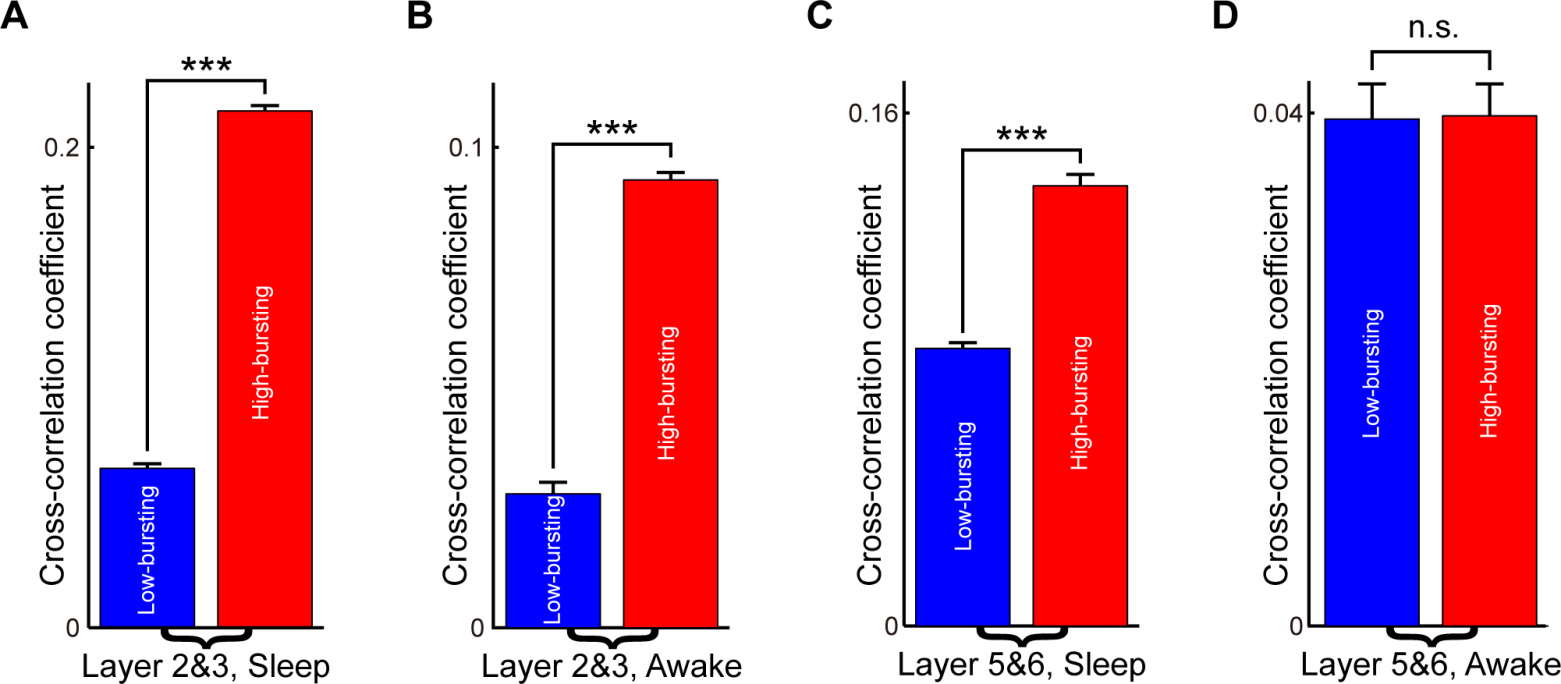
Layers 2&3 and 5&6 RSC principal cell subtypes showed distinct characteristics under cross-correlation analysis. **(A)** Cross-correlation coefficients of RSC layers 2&3 principal cell sub-populations during sleep periods. **(B)** Cross-correlation coefficients of RSC layers 2&3 principal cell sub-populations under the quiet awake state. **(C)** Cross-correlation coefficients of RSC layers 5&6 principal cell sub-populations during the sleep periods. **(D)** Cross-correlation coefficients of RSC layers 5&6 principal cell sub-populations under the quiet awake state. Error bars indicate s.e.m.; ns, nonsignificant (P > 0.05), ***P < 0.001.

To further investigate the properties of these subtypes of RSC excitatory principal cells, we measured the relationship between their spike activities using cross-correlation analysis. Layers 2&3 and 5&6 RSC principal cell subtypes showed distinct characteristics under cross-correlation analysis of their spike activities (see Methods). As shown in Fig 8A and 8B, in layers 2&3 of RSC, the high-bursting RSC principal cells exhibited significantly higher mean cross-correlation coefficient than the low-bursting RSC principal cells during sleep (Fig 8A, 0.214±0.003 vs. 0.066±0.003, ***p***<1E-145, two-sample *t*-test) and the quiet awake (Fig 8B, 0.094±0.004 vs. 0.028±0.003, ***p***<1E-29, two-sample *t*-test) periods.

For the two RSC layers 5&6 principal cell sub-populations (Fig 8C), the mean cross-correlation coefficient of the high-bursting RSC principal cells was significantly higher than the low-bursting RSC principal cells during the sleep period (0.137±0.007 vs. 0.087±0.004, ***p***<1E-7, two-sample *t*-test). However, there was no statistically significant difference between the high-bursting RSC principal cells and the low-bursting RSC principal cells in layers 5&6 during the awake period (Fig 8D, 0.040±0.006 vs. 0.040±0.005, ***p*** = 0.98, two-sample *t*-test).

### Profiling RSC excitatory principal cells under ketamine-induced anesthetized state

Since the ISICA-classified RSC layer 2&3 and 5&6 excitatory principal cells possess fundamental differences in intrinsic spike properties, we asked whether these subtypes may react differently to ketamine., a non-competitive NMDA receptor antagonist, can induce an anesthetic state referred to as "dissociative anesthesia,” that is, the patient is incapable of associating the input of afferent stimuli, and integrating information and signals to the conscious mind are reduced or blocked [46,47]. Dissociative anesthesia produced by ketamine has been postulated to be a result of reduced activation in the thalamocortical structures and increased activity in the limbic system and cortex [2]. To examine this, we investigated how the high-bursting and low-bursting excitatory principal cells reacted to ketamine-induced anesthesia. We compared the response characteristics of layer 2&3 and 5&6 excitatory principal cell subtypes between the quiet awake state and the ketamine-induced anesthetized state by measuring mean firing rates, burst indexes, the dynamic changes of the ISIHs, and power-density relationships.

**Fig 9:**
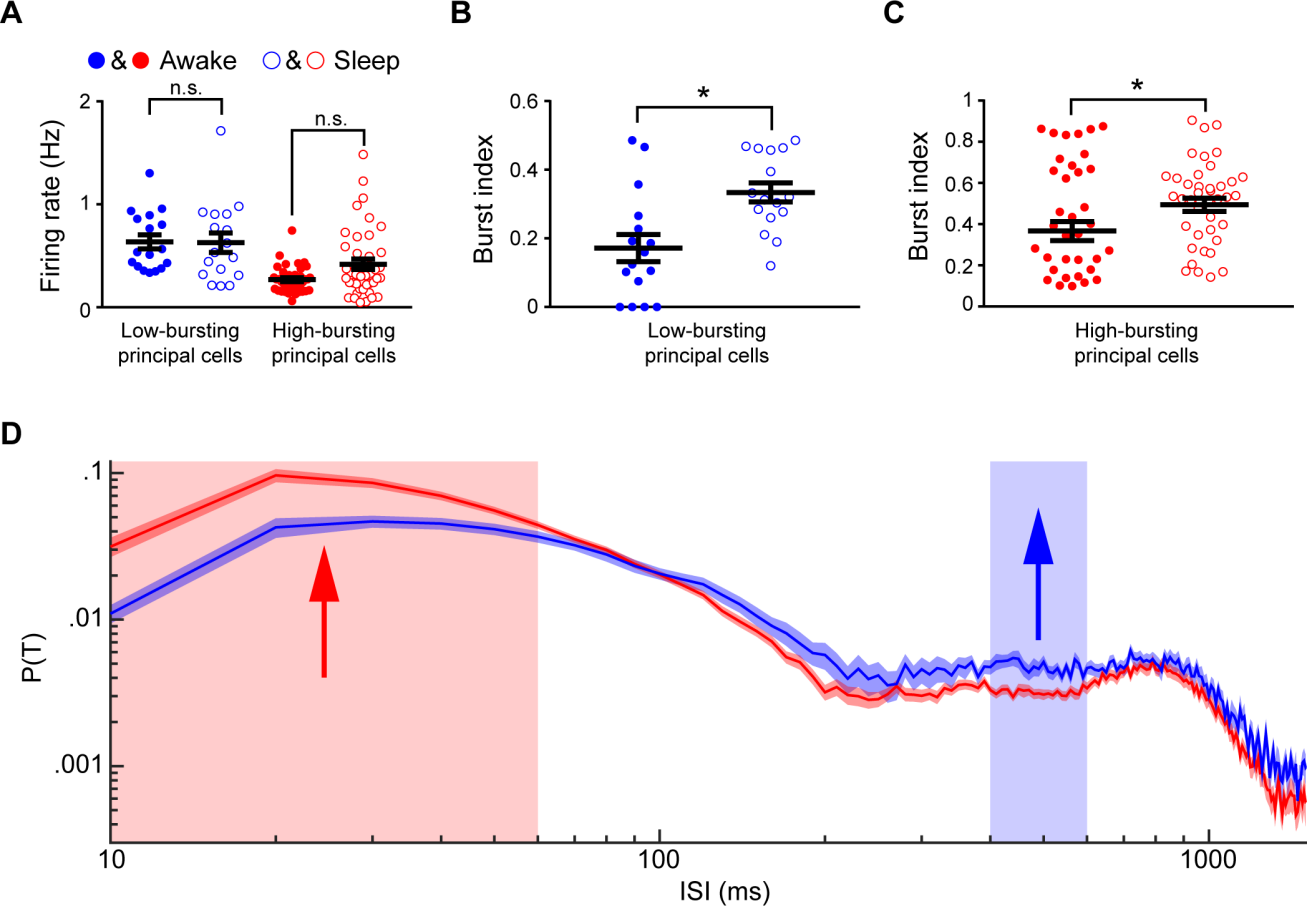
Principal cell subtypes in layer 2&3 showed significant differences upon ketamine-induced anesthesia. **(A)** Mean-firing rates of two-layer 2&3 principal cell subtypes during the quiet awake and anesthetized states. **(B)** Burst index of the low-bursting layer 2&3 principal cell subtypes during the quiet awake and anesthetized states. **(C)** Burst index of the high-bursting layer 2&3 principal cell subtypes during the quiet awake and anesthetized states. **(D)** ISIHs of two-layer 2&3 principal cell subtypes under the anesthetized state. Arrows and the colored regions in **D** showed that the significant differences between two principal cell subtypes. Error bars indicate s.e.m.; ns, nonsignificant (P > 0.05), *P < 0.05, **P < 0.01.

As shown in Fig 9A, there was no difference in the mean firing rates of the low-bursting and high-bursting layer 2&3 excitatory principal cell subtypes under these two states (Low-bursting cells: 0.64±0.07 (awake) vs. 0.63±0.10 Hz (anesthetized), ***p*** = 0.99. High-bursting cells: 0.27±0.02 (awake) vs. 0.42±0.05 Hz (anesthetized), ***p*** = 0.07. One-way ANOVA with multiple comparisons) Furthermore, the low-bursting layer 2&3 excitatory principal cells had significant higher firing rates then the high-bursting layer 2&3 excitatory principal cells under both quiet awake and anesthetized states (awake: ***p***<0.0001 and anesthetized: ***p*** = 0.047, one-way ANOVA with multiple comparisons). Surprisingly, the burst index of these two-layer 2&3 excitatory principal cell subtypes was increased upon ketamine treatment (Fig 9B and 9C, low-bursting layer 2&3 excitatory principal cells: 0.19±0.05 vs. 0.37±0.03, ***p*** = 0.012, high-bursting layer 2&3 excitatory principal cells: 0.37±0.04 vs. 0.49±0.03, ***p*** = 0.019, Kolmogorov-Smirnov test). Moreover, as shown in Fig 9D, the high-bursting layer 2&3 excitatory principal cells exhibited significantly higher firing probabilities at 10~60ms ISIs than those of the low-bursting layer 2&3 excitatory principal cells, while the low-bursting layer 2&3 excitatory principal cells had significantly higher firing probabilities at the ISI range of 400~600ms.

**Fig 10:**
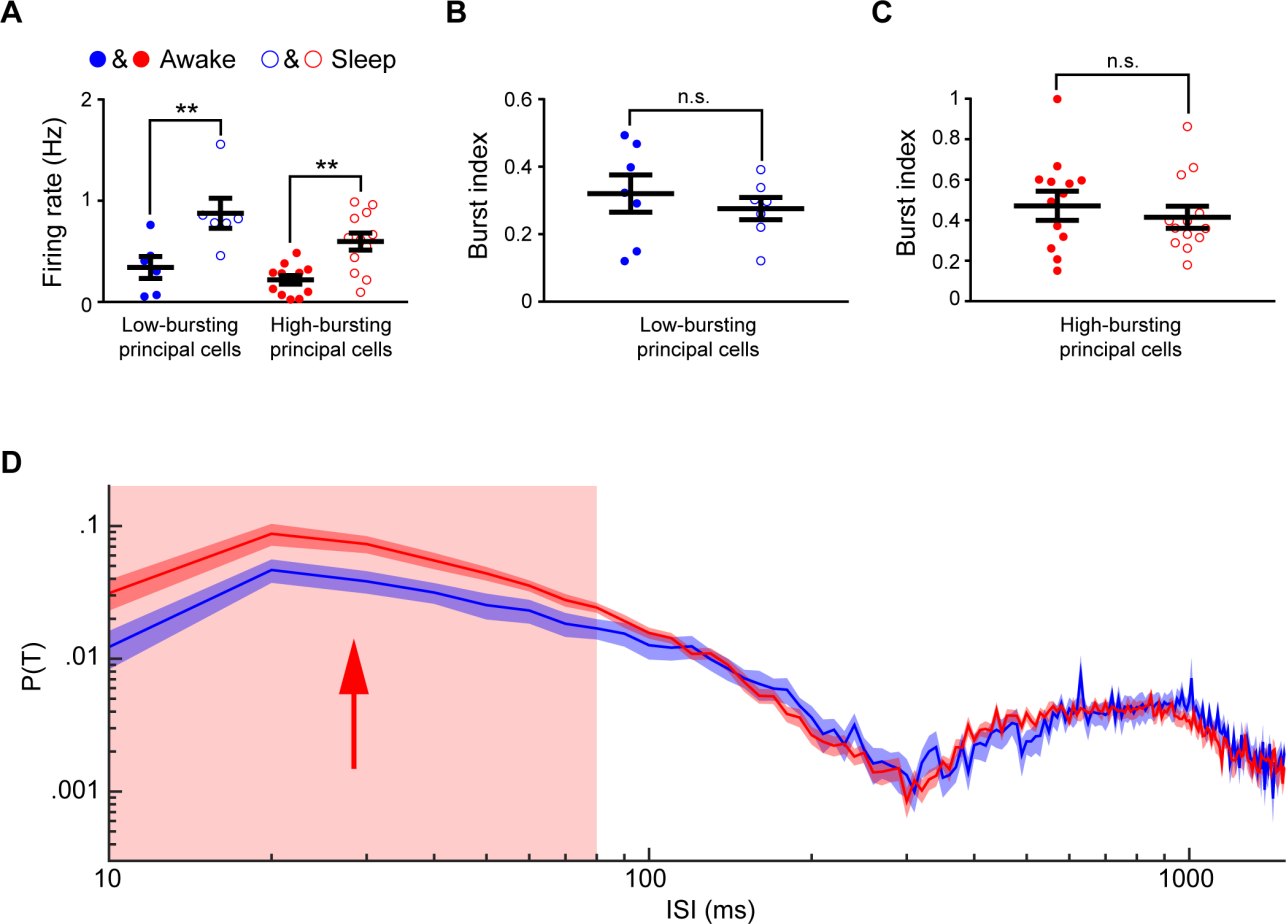
Principal cell subtypes in layer 5&6 showed significant differences upon ketamine-induced anesthesia. **(A)** Mean-firing rates of two-layer 5&6 principal cell subtypes during the awake and anesthetized states. **(B)** Burst index of the low-bursting layer 5&6 principal cell subtypes during the quiet awake and anesthetized states. **(C)** Burst index of the high-bursting layer 5&6 principal cell subtypes during the quiet awake and anesthetized states. **(D)** ISIHs of two-layer 5&6 principal cell subtypes under the anesthetized state. Arrows and the colored regions in **D** showed that the significant differences between two principal cell subtypes. Error bars indicate s.e.m.; ns, nonsignificant (P > 0.05), *P < 0.05, **P < 0.01.

We then conducted the same analyses for the two subtypes of layer 5&6 excitatory principal cells. As shown in Fig 10A, both of these two excitatory principal cell subtypes showed significantly higher firing rates upon the ketamine treatment (the low-bursting layer 5&6 excitatory principal cell subtypes: 0.34±0.11 vs. 0.87±0.15 Hz, ***p*** = 0.007, the high-bursting layer 5&6 excitatory principal cell subtypes: 0.22±0.04 vs. 0.59±0.19 Hz, ***p*** = 0.007, one-way ANOVA with multiple comparisons). Interestingly, the burst indexes of the layer 5&6 cells under quiet awake and anesthetized states showed no differences for both subtypes (Fig 10B: ***p*** = 0.58; Fig 10C: ***p*** = 0.29, Kolmogorov-Smirnov test). However, the firing probabilities of the high-bursting layer 5&6 excitatory principal cells were significantly higher than those of the low-bursting layer 5&6 excitatory principal cells at 10~80ms ISIs (Fig 10D).

**Fig 11:**
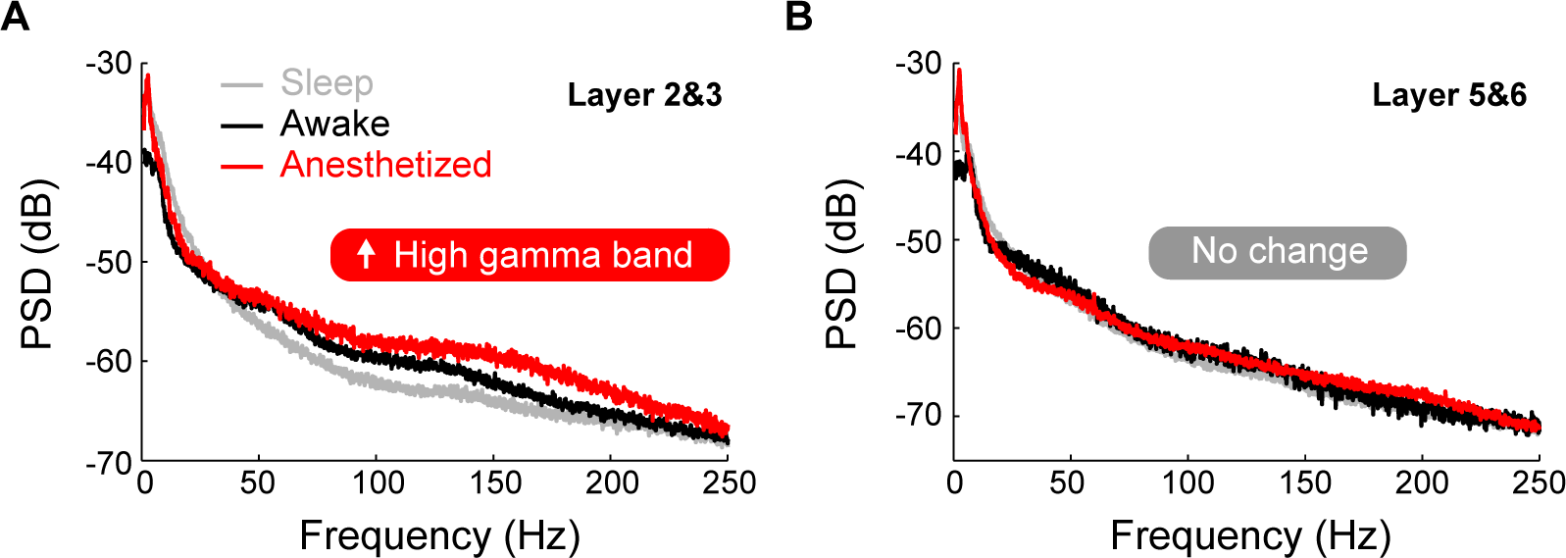
Average local field potential power spectra under the quiet awake, sleep and ketamine-induced anesthetized states. **(A)** Layer 2&3. **(B)** Layer 5&6. Gray curves, black curves and red curves represent the average local field potential power spectra under the quiet awake, sleep, and ketamine-induced anesthetized states.

Power spectral density analysis further conformed that there were distinct dynamics of the local field potential (LFP) between layer 2&3 and layer 5&6 under different brain states. As shown in Fig 11, average LFP power spectra were calculated for layer 2&3 and layer 5&6 under the quiet awake state, the sleep state, and ketamine-induced anesthesia. Compared with the LFP power spectra under the quiet awake state and the sleep state, the anesthetized state showed significantly higher power at the high gamma frequency band (120–200 Hz) in layer 2&3 (Fig 11A, ***p***<0.01, One-way ANOVA with multiple comparisons). Interestingly, there was no significant difference between the LFP power spectra under these three states in layer 5&6 (Fig 11B).

These experiments demonstrate that ISICA-classified cells in both layer 2&3 and 5&6 of RSC are differentially affected by ketamine-induced anesthesia.

## Discussion

In the present study, we characterized how cells in layers 2&3 and layers 5&6 of the RSC behave during conscious quiet awake, unconscious sleep periods, and ketamine-induced anesthesia. In each of the recorded layers, we uncovered multiple excitatory principal cell subtypes, namely high-bursting and low-bursting excitatory principal cells. The membership of these sub-populations remained identical during quiet awake and sleep periods. Importantly, during ketamine-induced anesthesia, these subtypes exhibit different dynamics, which is consistent with the notion that these RSC cells may play a different role in gating information integration essential for encoding declarative memory and knowledge.

Intriguingly, we were able to uncover high-bursting and low-bursting excitatory principal cells within both layers 2&3 and 5&6 of the RSC. It is of notable interest to reveal what specific functions these sub-populations may underlie. Interestingly, throughout the CA1 and subiculum, pyramidal cells have been uncovered that possess regular spiking patterns (i.e. later-bursting cells) and also bursting patterns (i.e. early-bursting cells) [48]. Additionally, in the subiculum, it has been reported that bursting cells are activated during sharp-wave associated ripples (SWRs), whereas regular firing cells are inhibited [49]. Because SWRs are known to be important for learning spatial memory tasks, it is possible that burst firing cells encode spatial content, whereas the regular firing cells may communicate non-spatial information [49]. Within the dentate gyrus, it has been demonstrated that induction of mossy fiber LTP depends on spike patterns that occur in high-frequency bursts [50]. Also, in a subset of cells, these supra-threshold bursts occurred during the sampling phase of a delayed nonmatch-to-sample task, and if this bursting occurred later, performance on the task was disrupted [50]. It is conceivable that identification of two distinct excitatory cell sub-populations in the RSC may enable future *in vivo* studies of the circuitry mechanisms related to synaptic plasticity and network oscillations.

Notably, the two subtypes of excitatory principal cells in both layers 2&3 and 5&6 remained distinct during different states, namely quiet awake and sleep periods. This preservation is intriguing and is reminiscent of our findings in the CA1 [45]. During the awake state, the brain is thought to be optimized for encoding memories, whereas memory consolidation is thought to be enhanced during sleep [51]. Interestingly, because the RSC is thought to function as a long-term memory storage site, it is of notable interest to uncover how excitatory principal cell sub-populations in this region may contribute to these cognitive functions. Distinct bursting activities of these two types of cells during the wakeful and sleep stages may suggest the possible conservancy of such dynamics related to learning as well as to consolidation of spatial and fear memory traces [52–55]. This will be addressed in future experiments.

The RSC possesses distinct laminar organization and importantly, receives a variety of excitatory and inhibitory projections from various brain regions [56,57]. For example, layer 1 of the RSC receives GABAergic projections from the hippocampal CA1 region and from the subiculum, which may influence both apical dendrites from excitatory cells residing in both layer 2 and deeper layers as well as parvalbumin (PV)-positive dendrites found in layer 2 [57–59]. Furthermore, layer 3 receives an excitatory projection from the subiculum [57]. Additional investigations are necessary to determine the exact postsynaptic targets of these projections [57]. How these projections influence the activity patterns of high-bursting and low-bursting excitatory principal cell subtypes in layers 2&3 and 5&6 remain for further investigation. Strikingly, within layers 2 and 3 of the RSC, a group of small excitatory cells have been shown to have a late-spiking property that is a result of A-type potassium and delayed rectifier channels [60]. It would be necessary to investigate how high-bursting or low-bursting excitatory principal cell populations uncovered in layers 2&3 might relate to these late-spiking excitatory cells. It is worthy to note that the high bursting excitatory principal cells in layers 2&3 of RSC exhibited significantly higher cross-correlation coefficients than the low-bursting RSC excitatory principal cells in the same layer during the quiet and sleep periods. In contrast, the high bursting RSC excitatory principal cells RSC of layers 5&6 was significantly higher than the low bursting RSC excitatory principal cells during the sleep period, but without any significant difference during the quiet awake period. This differential effect suggests that the high-bursting excitatory principal cells in layers 2&3 are likely to be distinct from those cells in layers 5&6, which may be due to differential regulation by local interneuron modulation or intrinsic biochemical and physiological properties.

Given the fact that many visual responses in the primary visual cortex have been studied under anesthesia, it will be interesting to investigate whether and how RSC cells are altered by ketamine or other pharmacological agents. For example, non-competitive NMDA receptors antagonists, MK-801, and ketamine have been reported to induce c-Fos expression in the RSC [61,62]. Interestingly, during ketamine-induced anesthesia, ISICA-classified excitatory principal cells in both layers 2&3 and layers 5&6 showed distinct dynamics. This may reflect different NMDA receptor subunit compositions on these excitatory principal cell sub-populations. Of notable interest, it has been observed that layer 2 cells of the entorhinal cortex (EC) display two discrete NMDA conductances, which suggests that these cells may express at least two different types of NMDA receptors [63]. Further studies are required to investigate whether similar phenomena occur in the RSC. Also, it is possible that the differential responses seen in both layers 2&3 and layers 5&5 may result from non-NMDA receptor mechanisms, as ketamine also acts on nicotinic, muscarinic, opioid, and various voltage-gated receptors [64]. For example, in layer 2 cells of the EC, muscarinic modulation of the firing and oscillatory properties of these neurons has been observed, which indicates a role for the cholinergic system in dynamically affecting the activity patterns of those cells [65]. How these various types of receptors are differentially expressed and may potentially contribute to distinct excitatory principal cell sub-populations in the RSC has yet to be explored.

Our discovery of differential responses of these two distinct RSC excitatory principal cell populations to ketamine raises interesting questions as to how they might be involved in health and diseases such as schizophrenia, which is known to produce visual hallucinations [66]. In addition, the RSC is known to contain a variety of histologically distinct excitatory cell types, including pyramidal and stellate cells [67–70]. It would interesting to complement our ISICA method with Cre/lox-mediated neurogenetic methods [71] to further investigate whether our uncovered excitatory principal cell subtypes are comprised entirely of pyramidal cells, stellate cells, or discrete variations of their subtypes. It would also be noteworthy to investigate whether the excitatory principal cell sub-populations in the RSC receive distinct projections from different subcortical or cortical regions. Similar findings have been reported in the hippocampal CA1 [72,73]. Future investigations will be necessary to explore this in the RSC.

Interestingly, layer 2&3 and layer 5&6 displayed distinct dynamics of their LFP during different brain states. In layer 2&3, LFP power spectra showed significantly higher power at the gamma frequency band compared with the quiet awake and sleep states. In contrast, there was no significant difference between the LFP power spectra under these three states in layer 5&6. Oscillations in the gamma frequency range are integral in memory storage and retrieval, sensory processing, and attentional selection [74]. Importantly, changes in oscillatory activity might are thought to be related to the pathophysiology of many psychiatric and neurological diseases. For example, aberrant gamma-frequency oscillations have been reported in schizophrenic patients [75]. Interestingly, ketamine can induce hallucinations and paranoia comparable with the positive symptoms of schizophrenia and behaviors that are reminiscent of the negative symptoms [66]. Our results show that ketamine induced higher gamma oscillations in superficial layers of the RSC, but not deeper layers. Within the hippocampus, the generation of independent and layer-specific gamma oscillations is thought to be explained by firing of GABAergic interneurons and selective axonal targeting [76]. Perhaps these layer-specific differences in RSC LFP can be explained by the activity of GABAergic interneurons within these layers [56,59,77]. Coordinated activity of excitatory principal cells and GABAergic interneurons (particularly PV-positive) is thought to govern the synchronization of gamma oscillations [78]. Notably, administration of phencyclidine, a non-competitive NMDAR antagonist, resulted in increased PV-immunoreactivity in the RSC [79]. Thus, it is possible that PV-positive interneurons within specific layers of the RSC are differentially affecting high gamma oscillations. This present study only characterized the activity of excitatory principal cells; however, future experiments will investigate the effect of ketamine on the dynamic activity patterns of inhibitory interneurons within the RSC. Additionally, it is possible that the layer-specific differences in LFP result from differences in axonal targeting between these layers [57–59]. Future experiments are required to delineate the contribution of these projections to ketamine-induced high gamma oscillations within the RSC. Overall, these results suggest that the effects of ketamine may be partially mediated by high-frequency activity in the superficial layers of the RSC.

In summary, by combining our ISICA method and *in vivo* recordings, we uncovered and profiled distinct sub-populations of excitatory principal cells and LFP within both layers 2&3 and layers 5&6 of the RSC during both quiet awake, sleep, and ketamine-induced unconscious states. This spike dynamics-based computational classification will be valuable to better examine how distinct neuronal subtypes in the RSC underlie specific memory engram or behavioral outcomes, especially as they relate to ketamine-induced psychosis.

## Online methods

### Ethics statement

All animal work described in the study were carried out in accordance with the guidelines laid down by the National Institutes of Health in the United States, regarding the care and use of animals for experimental procedures, and was approved by the Institutional Animal Care and Use Committee of Augusta University (Approval AUP number: BR07-11-001) and Banna Biomedical Research Institute Animal Care and Use Protocol BRI-2458.

### Surgeries and in vivo recording in the RSC region

The RSC units were recorded using a 32-channel tetrode recording array. The 32-channel electrode consisted of one independently moving bundle of 8 eight tetrodes in a linear array (Lin et al., 2006). One 36-pin connector array was positioned and secured with epoxy glue (5-minute epoxy gel, ITW Polymers Adhesives North America, Danvers, MA) to one side of the microdrive base. A linear array of 8 segments of polyimide tubing (inner diameter 99.9μm, outer diameter 167μm, Polymicro Technologies, Phoenix, AX) was attached to one movable screw nut on the base of the microdrive with glue. To construct the tetrodes, a folded piece of four wires (90% platinum, 10% iridium, 13μm, California Fine Wire Company, Grover Beach, CA) were twisted together and were secured with a low heat source. By moving the tips of the free ends of the tetrodes over an open flame, the insulation was removed. Each tetrode was placed through one of the polyimide tubes and after each was correctly positioned, the wires were glued to the polyimide tubes. The free end of each tetrode was wrapped around the connector pins and was individually soldered to their respective pins. A reference wire was then soldered to the final four pins on each end of the connector. Finally, the connector pin array was coated with glue and the looped tetrodes were glued to the base of the microdrive.

Wild-type B6BCA/J mice were given access to food and water in their cages and were handled for several days before the surgery to reduce the possible stress of human interaction. Before the surgery, the mouse was given an i.p. injection of 60mg/kg ketamine (Bedford Laboratories, OH) and 4mg/kg Dormitor (Pfizer Animal Health, NY). A stereotaxic frame was used to immobilize the head of the mouse and an ocular lubricant was used to coat the eyes of the mouse. The hair above the surgery site was removed and Betadine solution was applied to the surface of the skin before an incision was made along the midline of the skull. Hydrogen peroxide was placed onto the surface of the skull so that bregma could be visualized. The correct position for implantation was then marked, and four holes for screws were drilled and subsequently the screws were placed in these holes with a reference wire being secured to one of the posterior head screws. Dental cement was then used to fix the screws and a hole for the linear tetrode array was drilled. The dura was removed and the stereotaxic device was used to lower the array into the RSC. To record in layers 2&3, linear electrode arrays were implanted at 1.2 mm lateral to bregma and 2.5mm posterior to bregma on the right side at an angle of 45°. Layers 5&6 neurons were recorded by targeting linear electrode arrays to 0.4 mm lateral to bregma and 2.5mm posterior to bregma on the right side. Paraffin was used to fill the gaps surrounding the tetrodes and the microdrive was secured in position with dental cement. The reference wire from the connector pin array was soldered to that of the posterior head screw, and the copper mesh was used to surround the microdrive. The mouse was then injected with 2.5mg/kg Antisedan and placed in its home cage. Mice were allowed to recover for approximately one week before experiments began. All recordings were carried out while the mice were located in their home cages. Following the surgery, mice were monitored every 30 minutes for 3 hours. After all recording experiments were completed, mice were euthanized with an overdose of isoflurane. The positions of electrodes were then verified by brain sectioning and histological staining (Kuang et al, 2010).

To produce ketamine-induced anesthesia, the animals were then injected with a 60 mg/kg ketamine and 4mg/kg Domitor cocktail mixture via *i*.*p*., and the animals lost the righting reflex in a few minutes. Neural spike activities were recorded for at least 50 minutes under the anesthetized state. Neural spike data recorded from the fully anesthetized state starting from 10 to 45 minutes after the ketamine injections were selected for the present analysis.

### Data processing and spike sorting

The neuronal activity was recorded by a Plexon multi-channel acquisition processor system. The recorded spike activities were collected as described previously [80,81]. The well-separated neurons were assessed by “Isolation Distance” and “*L*_*ratio*_”. In the present analysis, neurons whose “Isolation Distance” >15 and “*L*_*ratio*_” <0.7 were selected for further analysis [80,81]. 215 well-separated neurons were recorded from RSC layer 2&3 of nine mice, 262 well-separated neurons were recorded from RSC layer 5&6 of 13 mice.

### Assessing quiet awake and sleep states

Quiet awake episodes were manually assigned when the mice were awake and immobile. To identify slow-wave sleep, local field potentials were first band-pass filtered in theta band (4–12 Hz) and delta band (1–4 Hz), and then the ratio of the power in theta band to delta band was calculated. Two criteria were applied to extract the slow-wave sleep state: (1) Duration of an epoch was longer than 5s, and (2) The ratio of the power during an epoch was greater than mean + 5SD. For each mouse, the quiet awake and SWS states were recorded for at least 15 minutes.

### Inter-Spike Interval Classification Analysis (ISICA)

The detailed procedure of ISICA has been previously described (Li et al. 2015). There were four computational analysis steps in ISICA: 1) feature extraction from spike dynamic patterns; 2) pre-clustering analysis on these extracted features; 3) performing classification and clustering analyses to examine neuronal feature subtypes in different dimensions; 4) determining the optimal dimension from which the best classification/clustering can be selected in an unbiased manner. Specifically, two parameters, shape parameter *k* of the Gamma distribution model and coefficient of variation *c*_*v*_, were employed to characterize the inter-spike interval properties of each neuron; the D’Agostino and Pearson omnibus normality test was used to determine if there were multiple sub-populations in the recorded dataset; the neuron sub-populations were then uncovered by the unbiased clustering analysis (combining *k*-means clustering and the jump method). Finally, a bootstrap analysis was employed to verify that the recorded dataset was indeed best represented by the uncovered sub-populations.

### Unbiased clustering analysis based on the k-means clustering and the “jump method”

Unbiased clustering analysis was conducted by combining the *k*-means clustering method and the “jump method”[82] to unbiasedly uncover neuron subtypes. In the present analysis, the distance measurement used by *k*-means clustering was the cosine distance, which was defined as one minus the cosine of the included angle between vectors. Each centroid was the mean of the points in that cluster. The “jump method” was introduced to determine the optimal number of clusters within the data. Since the *k*-means partitioning may depend upon the starting points used, the *k-*means algorithm is repeated a number of times (10,000 times) with different starting conditions, and a mean distortion for each prescribed value of *k* is obtained. A distortion curve is then generated by plotting the mean distortion as a function of *k*. The distortion tends to decrease as the number of clusters is increased, and this is transformed into an increase by raising the distortion to a negative power. Because the distortion drops when the correct number of clusters is used and remains roughly constant when even more clusters are employed, the transformed distortion exhibits a sudden increase, or jump, at the optimum value of *k*. If one examines the size of the jumps in the transformed distortion, the largest jump is thereby an indication of the proper number of clusters. Thus, using spike data from the quiet awake and sleep states, the optimal number of clusters was unbiasedly chosen by the “jump method.” We also checked our clustering results with another 10,000 repeats, the clustering results of two different cell types in RSC layer 2&3 and 5&6 were preserved with probabilities of 98.45% and 98.63%, respectively. The cosine similarity gives the most robust clustering results (>98.4%), while the Euclidean distance and Mahalanobis distance also produce acceptable clustering results (>87.2% for Euclidean distance and >81.1% for Mahalanobis distance).

We used cluster index to measure the quality of clustering results. Cluster index[73] is defined as the ratio of the sum of the square distances from each point to its cluster center and the sum of the square distances from each point to the overall mean. A million cluster index values were calculated by repeatedly drawing samples from a single multivariate Gaussian distribution, and the ***p*** value was defined as the likelihood that the simulated data had a cluster index greater than the experimental data. The simulated results showed that the spike data were best represented by two clusters (***p***<1E-10 for all analysis).

A Gaussian mixture model analysis was performed for quantifying the overlap between the clusters. For all our clustering results, the average probabilities of each point belonging to the other cluster were < 5% (Table 1).

**Table 1:** Average probability of each point belonging to the other cluster (%).

	Layer 2&3				Layer 5&6			
	Awake		Sleep		Awake		Sleep	
	Cluster 1	Cluster 2	Cluster 1	Cluster 2	Cluster 1	Cluster 2	Cluster 1	Cluster 2
k	1.62±0.23	4.40±0.16	4.64±0.19	2.04±0.34	1.15±0.21	3.13±0.16	4.24±0.07	0.86±0.16
Cv	0.09±0.01	0.06±0.01	0.05±0.01	0.07±0.01	0.11±0.01	0.04±0.01	0.05±0.01	0.05±0.00

### Hierarchical clustering analysis

Hierarchical clustering analysis was performed by using the customized MATLAB program which incorporated the MATLAB functions “pidst” and “linkage”. The “pdist” function calculates the distance between objects with the option of “cosine”. The “linkage” function takes the distance information generated by “pdist” and links pairs of objects that are close together into binary clusters. The linkage function then links these newly formed clusters to each other and to other objects to create bigger clusters until all the objects in the original data set are linked together in a hierarchical tree. The “dendrogram” function plotted the cluster trees.

### Inter-cluster distance vs. intra-cluster distance

The inter-cluster distance is the Euclidean distance from each point to the centroid of their assigned cluster, while the intra-cluster distance is the Euclidean distance from each point to the centroid of their non-assigned cluster. The inter-cluster distance of accurate clustering should be significantly higher than intra-cluster distance.

### Cross-correlation analysis

We calculated the Pearson correlation coefficients between spike trains of principal cells. A cell’s spike train was first converted into a vector using binsize of 500 ms, the pairwise spike correlations were then calculated among all neurons of each principal cell subtypes (using MATLAB function “corrcoef”).

### Local field potential spectral analysis

The local field potential power density was calculated in a range of 0.1–250.0 Hz with 0.1 Hz intervals for the datasets recorded in layer 2&3 as well as layer 5&6. The fast Fourier transform was applied to the EEG signal, the resulting frequency resolution was 0.1 Hz, and the frequency bins less than 1 Hz were discarded due to the sensitivity of these bins to noise.

### Test of statistical significance

In the present study, one-way ANOVA analysis and Tukey *post hoc* tests were conducted for the comparisons of multiple means. Student’s *t*-test was used here to assess whether two sets of data were significantly different from each other. In all figures that contained statistical significant test results, one asterisk denoted that the ***p***-value is in the range of 0.05–0.01, two asterisks denoted that the ***p***-value is in the range of 0.01–0.001, three asterisks denoted the ***p***-value is less than 0.001. Data were represented as mean ± S.E.M.
